# Verbal interaction pattern analysis in clinical psychology

**DOI:** 10.3389/fpsyg.2022.949733

**Published:** 2022-07-26

**Authors:** Jesús Alonso-Vega, Natalia Andrés-López, María Xesús Froxán-Parga

**Affiliations:** ^1^Faculty of Biomedical and Health Sciences, Universidad Europea de Madrid, Madrid, Spain; ^2^Department of Biological and Health Psychology, Universidad Autónoma de Madrid, Madrid, Spain

**Keywords:** process-based therapy, clinical psychology, pattern analysis, sequential analysis, verbal interaction, principle-based therapy

## Abstract

Recent developments in pattern analysis research have made this methodology suitable for the study of the processes that are set in motion in psychological interventions. Outcome research, based on the comparison between clinical results from treatment and control groups, has leveraged our empirical knowledge about the efficacy of psychological interventions. However, these methods of research are not precise enough for the analysis of these processes. On the contrary, pattern analysis could be a powerful tool to study moment-to-moment interactions typical of psychological interventions. This is methodology is relevant because clinical psychology is experiencing a paradigm shift from a protocol for syndrome perspective to a principle-based and person-centered intervention. This evidence-based, theory-grounded, and process-oriented paradigm of clinical intervention needs new research methods to thrive (i.e., pattern analysis). The analysis of the therapeutic relationship built into the verbal interaction between the clinician and the client is one of the cornerstones of this new era of research. So, the purpose of this article is three-fold: (1) to discuss the role of the verbal interaction pattern analysis in the clinical context to the development of the principle-based clinical psychology, (2) to analyze the patterns of verbal interaction in a clinical case, and (3) to compare the results using two different methods. To reach these purposes, using the observational methodology, we have coded the verbal interaction of 16 clinical sessions with a person diagnosed with a borderline personality disorder. We have analyzed the data using sequential analysis (GSEQ) and pattern recognition algorithms (i.e., T-Pattern detection). We have been able to detect typical patterns during different phases of psychological intervention (i.e., evaluation, explanation, treatment, and consolidation). Finally, the conceptual, methodological, and empirical implications of this study will be discussed within the realms of pattern analysis research and principle-based clinical psychology.

## Introduction

*Pattern* is a term that refers to a stable repetition of events that arise from specific circumstances. The recognition of patterns in the natural world has had an evolutionary impact on the animal species, it has allowed us to adapt to the environment by taking advantage of these regularities. Moreover, understanding how these patterns work has led us to control and predict these natural events. Recognition and analysis of patterns are scientific endeavors that have benefited from the efforts of multiple scientific fields (e.g., mathematical definitions, biological theories, etc.) and are useful to improve applied sciences (e.g., engineering, medicine, etc.). Furthermore, the development of new definitions of patterns or the application of existing methods of analysis to new areas could solve contemporary problems in several scientific disciplines, we believe that this is the case in clinical psychology. This paper aims to improve clinical psychology research using pattern analysis methods (e.g., T-pattern Analysis), to do that first we define the state of the art in clinical psychology research highlighting the current challenges, then we analyze the conceptual and practical opportunities that pattern analysis methods open to this field of research, and finally we conduct a proof-of-concept study.

Clinical psychology has reached an important stage of development in regards to the scientific agreement about the efficacy of psychological interventions for specific psychological problems (David et al., 2018; Van Agteren et al., 2021). Thanks to the evidence-based psychotherapy movement, this milestone has ended a historical debate within our field (Forte et al., 2014). While, it has set the opportunity to face new challenges and research questions, as well as analyze the flaws of the current clinical research system. For example, Paul’s classic question, “What treatment, by whom, is most effective for this individual with that specific problem, and under which set of circumstances, and how does it come about?” (Paul, 1969, p. 44), is not yet solved. To answer this question we need to implement conceptual, experimental, and applied changes to our clinical research and our validation systems (Tolin et al., 2015). Among these changes, we would like to emphasize the importance of clinical process research rooted in a functional perspective of behavior, experimentally validated processes, and the connection between intervention outcomes and the processes that could explain these behavioral changes (Hayes and Hofmann, 2018; Barnes-Holmes et al., 2020). Especially, two main trends are rising in clinical psychology, the call for personalized psychotherapy and the study of complex interactions between psychological problems, psychological interventions, and treatment outcomes (Hofmann et al., 2020; Wright and Woods, 2020; Mansueto et al., 2022). Due to the nature of these two trends, clinical research needs new methods and approaches to conduct idiographic studies in clinical psychology. There is a long tradition of idiographic research in clinical psychology (Kazdin, 1978; Hayes, 1981; Iwata et al., 1994), but the emphasis on this type of research is renewed (e.g., Molenaar, 2004; Beltz et al., 2016; Piccirillo et al., 2019; Kazdin, 2021). New professional standards for single-case methods (Tate et al., 2016; Horner and Ferron, 2022) and the development of new methods for the interpretation of these designs (Hedges et al., 2013; Kratochwill and Levin, 2014; Pustejovsky, 2018; Manolov et al., 2021) could help us to analyze the learning processes, that occurs at the individual level and are settled in motion by the psychologists’ procedures, that account for the clients change.

Specifically, in the psychological interventions with adults, these processes occurred within the verbal interaction between the psychologist and the client. Other important variables explain the therapeutic change of our clients (e.g., contextual, and cultural variables outside of the clinical session, setting variables, motivational variables, etc.), but the verbal interaction with the client is the main channel that psychologists have to implement their procedures (Follette and Bonow, 2009; Tsai et al., 2014; Virues-Ortega and Froxán-Parga, 2015).

The interest in the study of verbal interaction in clinical sessions from a functional perspective is present since the early 60 s (see Moore, 1991). This conceptual development helped to foster empirically-supported psychological treatments like the Functional Analytic Psychotherapy (Kohlenberg and Tsai, 1991) or the Acceptance and Commitment Therapy (ACT; Hayes et al., 1999; Hayes, 2004)^1^. Also, helped to develop coding systems for the observational study of the verbal interaction like the Functional Analytic Psychotherapy Rating Scale (FAPRS; Callaghan et al., 2008); the Multidimensional System for Coding Behaviors in Therapist-Client interaction (SiMCCIT; Zamignani, 2008) or the Verbal Interaction Categorization System in Therapy (Froxán-Parga et al., 2011; Alonso-Vega et al., 2022). Using these coding systems allowed to describe the verbal interaction between psychologist and client in clinical sessions (e.g., Froxán-Parga et al., 2016), to study the effects of it outside of the session (e.g., Lizarazo et al., 2015), to study the molecular learning processes that occur in this interaction (e.g., Busch et al., 2009), to analyze the interaction during specific techniques like cognitive restructuring (e.g., Calero-Elvira et al., 2013; Froxán-Parga et al., 2018), and to conduct experiments to study the verbal shaping during clinical sessions (Pardo-Cebrian et al., 2021). These works helped to analyze the basic principles of change in psychological interventions, however, they report methodological problems to study moment-to-moment interactions. For example, Busch et al. (2009) and Xavier et al. (2012) used transitional probabilities to study these interactions. Transitional probabilities inform us about the probability of an event (X) given other (Y), but this is a limited method to study the verbal interaction in psychological interventions because these probabilities reflect behavior frequencies in a particular session and are not comparable over sessions (Bakeman and Quera, 2011). Other, studies (see Calero-Elvira et al., 2013) opted to use sequential analysis to analyze these moment-to-moment interactions. Sequential analysis techniques help us to study patterns and temporal associations among behaviors within observational sessions (Bakeman and Quera, 2012), these techniques are based on the calculation of contingency indices for 2 × 2 tables so they are limited to two-term contingencies. Also, these sequential analyses, normally are used to confirm an expected sequential relationship between two events and are not used to explore patterns that are hidden from the observer’s eyes (Magnusson, 2005). Thus, more sophisticated pattern detection and analysis techniques could help us to detect hidden patterns of verbal interaction in clinical sessions and thus to have more precise data that enable us to better study the learning principles that could explain the client’s behavioral changes that are set in motion in the verbal interaction between the psychologist and the client. So, the application of the T-Pattern, using THEME software that allows us the automatic detection of temporal and sequential structures in observational data, could be useful to overcome the methodological limitations of previous studies of the verbal interaction analysis in clinical settings. Consequently, the purpose of this study is to analyze the patterns of verbal interaction in a clinical case using the THEME software and to compare the results with previously used analyses, as in Lapresa et al. (2013).

## Materials and methods

### Participants

For this observational study we had the participation of a 31-year-old client diagnosed with borderline personality disorder (BPD) for the last 8 years; and a 35-year-old clinical psychologist (a master’s degree in General Health Psychology and a master’s degree in Behavior Therapy). Both participants came from a public-funded Vocational Rehabilitation and Employment center (VR&E) in the Community of Madrid. The client has been referred to this center due to mood problems (i.e., emotional lability) and substance use problems that directly interfere with the client’s chances of accessing employment/training opportunities; and once obtained, problems in keeping his job or completing the required training. Also, this study involved the participation of two trained observers. Both observers are predoctoral students that have been trained in the same research group (i.e., the ACOVEO research group). Observer 1 and Observer 2 have, respectively, 4 and 2 years of experience working with the observational coding system used in this research and they helped in the development of it. Before recording the clinical session, the client and clinician have been informed about the use of the data and the purpose of the research. All participants have signed the study informed consent.

### Instruments

We used the Functional Coding System for Verbal Interaction in Clinical Contexts (Alonso-Vega et al., 2022) to code the verbal interaction between the client and the psychologists. This coding system, which focuses on the putative functions of the verbal behavior, has five coding categories for the clinician verbalizations: *Clinical Discriminative Stimulus* (CD) and *Instructional Discriminative Stimulus* (ID), *Conditioned Motivating Operation* (CMO), and *Positive Reinforcer* (R+) and *Aversive Stimulus* (AS). The observational system assumes that the verbal behavior of the client has a response function it is not established any specific categories in the observational system. To code the client’s verbal behavior in this case we used eight categories based on the response topography *Giving Information* (GI), *Asking for Information* (AI), *Following Instructions* (FI), *Not Following Instructions* (NFI), *Well-Being* (WB), *Discomfort* (D), *Target Behavior* (TB) and *Problem Behavior* (PB). See Table 1 for a brief description of the coding categories, also more details of the coding system are available in the additional materials.

**Table 1:** Brief description of the coding categories.

Coding categories	Abbreviation	Putative function	Example
Clinical Discriminative	CD	Antecedent stimulus that increases the probability of a response class. (e.g., to give clinical information)	“How do you feel about that?”
Instructional Discriminative	ID	Antecedent stimulus that increases the probability of a response class (e.g., to follow instructions)	“I want you to apply the breathing technique every night.”
Conditioned Motivating Operation	CMO	Antecedent stimulus that changes the reinforcing value of a consequent stimulus and changes the frequency of responses related with this consequent stimulus	“If you run daily, your situation will improve.”
Positive Reinforcer	R+	Consequent stimulus that increases the probability of an operant response with a positive contingency with it	“Very good! You have done great.”
Aversive Stimulus	AS	Consequent stimulus that decreases the probability of an operant response with a positive contingency	“I do not agree with what you have done.”

This table only displays a brief description of the clinician coding categories. Client coding categories are topographically based and they depend on each clinical case. Please see the additional materials for further details.

### Materials

Recordings of 16 clinical sessions were obtained through the camera installed on the VR&E psychologist’s computer. The recordings were sent to the ACOVEO research group and were treated following the protocol used in the research group in which the recordings are anonymized following the ethical and legal guidelines of the Organic Law 3/2018 on Personal Data Protection and guarantee of digital rights. These recordings were stored on external hard drives kept under lock and key in the group’s laboratory at the Autonomous University of Madrid.

The recording of the clinical sessions, the observation project, and the analysis of inter-observer reliability were carried out with *The Observer XT 12* observation software. The data analysis was done in *R* (RStudio Team, 2020) and *Microsoft Excel*. *GSEQ* (Bakeman and Quera, 2016) was the software used to conduct the sequential analysis of the data. We selected this software because it is generally employed in observational research (e.g., Santoyo et al., 2017; Brown et al., 2018); it was specifically used in previous research in the study of verbal interaction in clinical cases (Calero-Elvira et al., 2013; Pardo-Cebrian et al., 2021); and it was specially developed to calculate sequential patterns in observational data (Bakeman and Quera, 1995, 2016). Finally, we used *ThemeEdu* software (Pattern Vision, 2021) for automatic pattern detection. *ThemeEdu* was selected because it has been successfully applied in different research areas (i.e., neuronal interactions, behavioral interactions, etc.; Magnusson, 2020), but it was not employed in the study of verbal interactions in clinical settings. Both programs allowed us to conduct the data analysis described in the section below (see “Data Analysis”).

### Procedure

In this observational study, we used an intra-subject design with three different phases: Evaluation (EVA.), Treatment Phase 1 (T1), and Treatment Phase 2 (T2). These phases were not experimentally manipulated and have been divided considering the protocols and procedures of the VR&E center (i.e., EVA, first three sessions; TP1, 4–9 sessions; and TP2, 10–16 sessions). The division between the two treatment phases is arbitrary and responds to a need to divide the treatment into, at least, two phases to evaluate differences between various time points.

After the study, both observers received specific training in the observational instrument. The training process was completed when a stable reliability index (*k* > 0.70) was achieved while coding similar clinical sessions. These sessions were from the research team’s clinical sessions archive.

After training was completed, observer 1 individually recorded all treatment sessions. Observer 2, also individually, recorded 4 random sessions out of the 16 treatment sessions, representing 25% of the sample, which is above the usual 10% for studies of this type. The inter-observer reliability calculation was carried out after the end of the recording of both observers. One month after the end of the recording phase, observer 1 recorded again two randomly selected treatment sessions to allow the calculation of intra-observer reliability.

### Data analysis

The kappa coefficient (k) was used to calculate the inter-observer and intra-observer reliability of the records. Once the records were obtained, the rate per minute of each variable recorded in each session was calculated. Descriptive data (e.g., count, rate per minute, etc.) were obtained to allow a visual inspection of the variables and the patterns through time. To analyze the interaction between variables, we used two types of statistical analysis, which are part of the family of statistical tools for the sequential analysis of temporally distributed data. First, we calculated the Yule’s Q, a contingency index of 2×2 tables, using the GSEQ software (Bakeman and Quera, 1995, 2016). This index allows us to calculate the Lag +1 correlation between a given behavior and the one that follows it. The Lag-1 correlation tells us which behavior precedes the behavior we are analyzing. This index allows descriptive and analytical analysis of the association (Bakeman and Quera, 2016) and its scores can also be interpreted in the same way as Cohen’s r. To study the association between specific pairs of behaviors we calculated the adjusted residuals (*z*), which are a normalized index of the extent to which the values of the frequencies observed in each cell of the matrix deviate from their expected values: a value greater than 1.96 indicates that this behavior occurs significantly more than expected and, conversely, a value less than −1.96 implies that it occurs significantly less than expected by chance (*p* < 0.05; see Bakeman and Quera, 2011 for an advance mathematical description).

Moreover, we used a pattern recognition model *T-Pattern Model* (Magnús et al., 2016; Casarrubea et al., 2018), using *ThemeEdu* software (Pattern Vision, 2021). This model allows us to recognize patterns (*T-Patterns*) from observational data (*T-Data*) using the *T-Pattern* detection algorithm. Pattern detection works in a bottom-up fashion, from the data to the pattern detection. The *T-Pattern* is a hierarchical, multi-ordinal, and self-similar pattern type that comprises *m* ordered components (i.e., behavioral events), X_1.m_, recurring in a single discrete dimension, where each component is a *T-data* category (or pattern primitive, called event-type) or a *T-pattern* (Magnusson, 2020).

In this case, patterns of interaction between client and therapist have been detected in all three phases of observation. We used the pattern recognition default settings with some specifications. We have required that the patterns must be repeated at least three times in each session and all sessions of each phase, with this we want to make sure that the pattern is a characteristic of this phase not a characteristic of one of the sessions. Also, we have excluded the patterns made by the repetition of the same variable and the patterns made by an interaction of variables of the same subject, because we want to study the interaction between variables. Finally, we have required a maximum of 4 levels for the analysis of the interaction, because more than four levels could be interpreted as chains made of contingencies of two or three members.

To assess the effect of the treatment on the client’s behaviors we calculated the effect size, in this study we used the Non-overlap of All Pairs (NAP) which is an index focused on identifying the differences between two phases of a design (A and B; Parker and Vannest, 2009; Carter, 2013).

## Results

### Reliability

Inter-rater reliability is 0.71–0.86, and intra-rater reliability is 0.82–0.89. Both *k* values can be interpreted as very good and excellent reliability indexes (McHugh, 2012).

### Using SDIS-GSEQ

**Figure 1:**
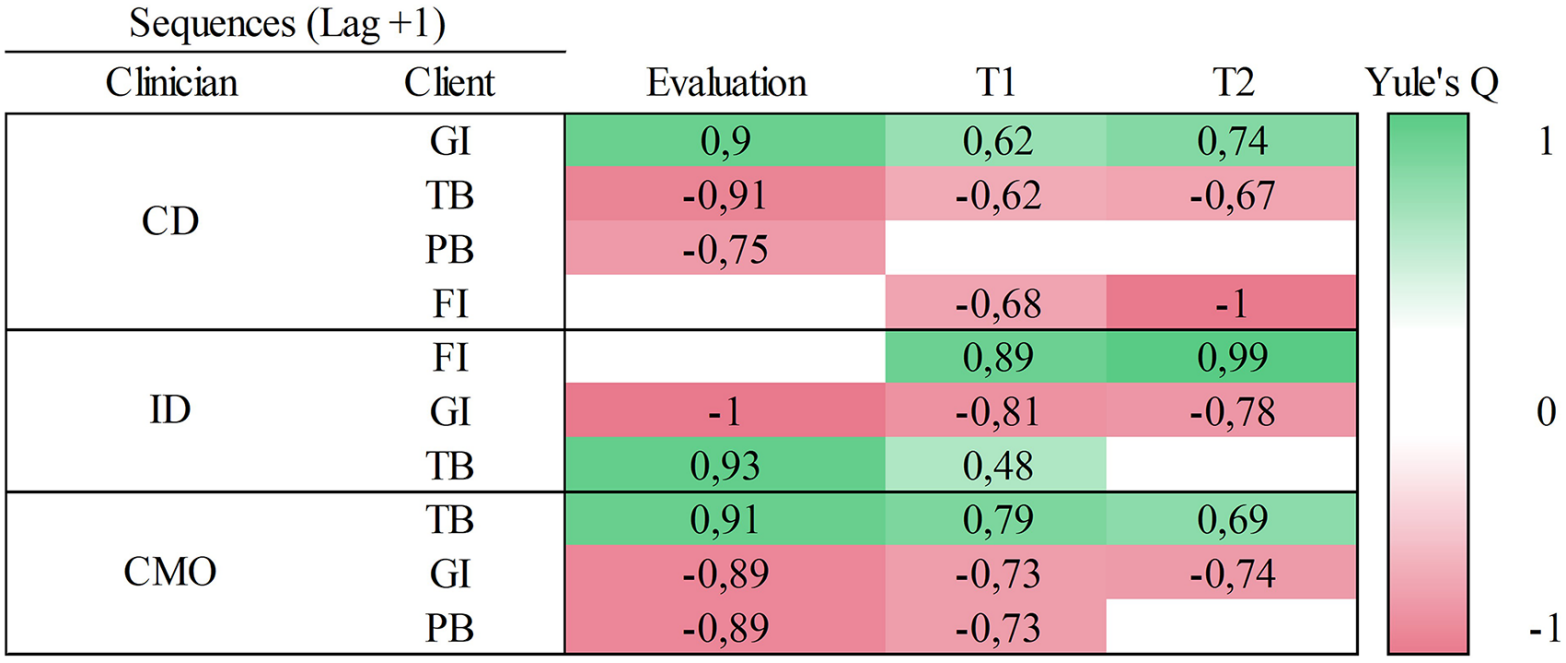
Antecedent sequential analysis using GSEQ. This figure displays sequential relationships between clinician’s behaviors and the client’s behaviors that have been followed by. Here the positive relationships are shown in green and indicate that these are significatively correlated behaviors (*Q* > 0; *p* < 0.01.). For example, CD was followed by GI in all treatment phases. Negative ones, in red, indicate that those behaviors were not observed together during the verbal interaction (*Q* < 0; *p* < 0.01). CD, Clinical Discriminative Stimulus; ID, Instructional Discriminative Stimulus; CMO, Conditioned Motivating Operation; GI, Giving Information; TB, Target Behavior; PB, Problem Behavior; FI, Following Instructions; Ti, Treatment phase 1; T2, Treatment phase 2.

**Figure 2:**
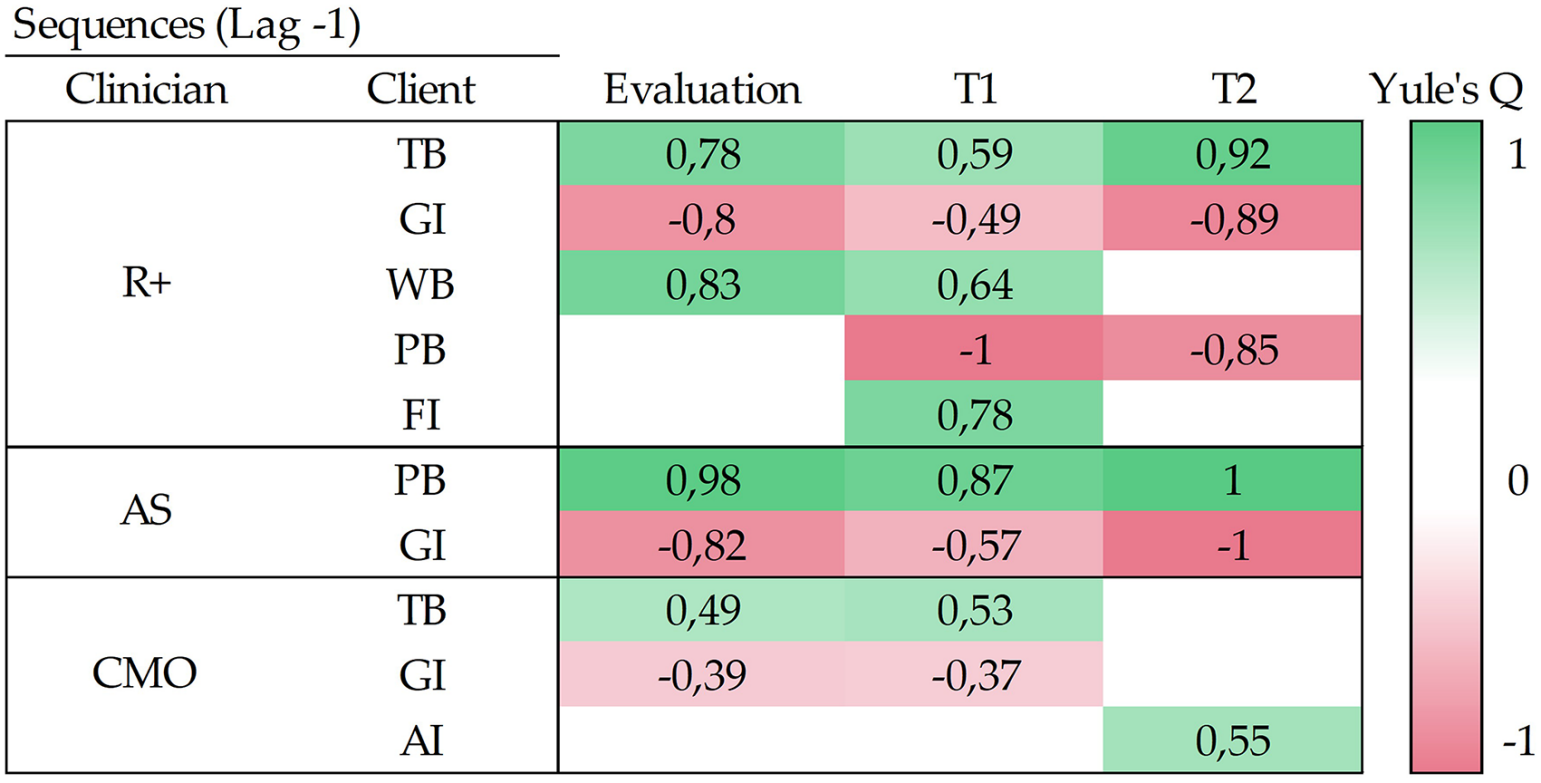
Consequent sequential analysis using GSEQ. This figure displays sequential relationships between client’s behaviors and the clinician’s behaviors that have been followed by. Here the positive relationships are shown in green and indicate that these are significatively correlated behaviors (*Q* > 0; *p* < 0.01). For example, TB was followed by R+ in all treatment phases. Negative ones, in red, indicate that those behaviors were not observed together during the verbal interaction (*Q* < 0; *p* < 0.01). R+, Positive Reinforcer; AS, Aversive stimulus; CMO, Conditioned Motivating Operation; GI, Giving Information; TB, Target Behavior; WB, Well-Being; Problem Behavior; FI, Following Instructions; AI, Asking for Information; Ti, Treatment phase 1; T2, Treatment phase 2.

Figures 1, 2 show the results of antecedent (Lag+1) and consequent (Lag-1) sequential analysis using GSEQ. All displayed correlations are significant (*p* < 0.01), positive correlations (*Q* > 0) are painted in green, and negative correlations (*Q* < 0) are painted in red. Data indicate that there is a positive correlation between *Clinical Discriminative Stimulus* with the client’s *Giving Information* behavior in all phases of the case (see Figure 1). *Following Instructions* correlates positively with the occurrence of *Instructional Discriminative Stimulus*, but this only occurs in the treatment phases (i.e., it is not found in the assessment phase); *Following Instructions* correlates positively with *Target Behaviors* during Evaluation and T1. Finally, Figure 1 also shows a significant positive correlation between *Conditioned Motivating Operation* and *Target Behaviors* in all phases.

The consequent sequential analysis indicates a positive correlation between *Positive Reinforcers* and *Target Behaviors, Well-being Verbalizations, Following Instructions, Aversive Stimuli,* and *Problem Behavior.* Also Conditioned Motivating Operations were weakly correlated with *Target Behaviors* and *Asking for Information.*

### Using THEME

Tables 2, 3 show the frequency of two-, three- and four-term patterns in each part of the clinical case. For example, Table 2 displays a strong relationship between *Clinical Discriminative* and *Giving Information* in all phases of the clinical case, this pattern repeats 888 times through different clinical sessions. Also, there are some patterns especially repeated in the early stages of the treatment (e.g., *Clinical Discriminative* and *Discomfort*, *Instructional Discriminative* and *Target Behavior*), and in treatment phases (e.g., *Clinical Discriminative* and *Wellbeing, Clinical Discriminative,* and *Target Behaviors*). Moreover, we have detected patterns increasing through the phases (e.g.*, Conditioned Motivating Operation* and *Target Behaviors*, *Target behaviors* and *Positive Reinforcers*).

**Table 2:** Two-term pattern analysis using THEME.

Pattern		Number of patterns		
*Clinician*	*Client*	Evaluation	T1	T2
CD	GI	221	424	243
CD	WB	–	24	13
CD	TB	–	–	81
CD	AI	–	–	19
CD	PB	–	–	27
CD	D	10	–	–
ID	TB	15	19	–
CMO	TB	54	87	104
CMO	AI	–	–	14
*Client*	*Clinician*	Evaluation	T1	T2
TB	CMO	41	–	25
TB	R+	65	63	97
WB	DC	–	–	16
WB	R+	11	21	–
GI	R+	19	56	–
PB	AS	–	14	14
FI	R+	–	13	–
AI	CMO	–	–	10

CD, Clinical Discriminative Stimulus; ID, Instructional Discriminative Stimulus; CMO, Conditioned Motivating Operation; R+, Positive Reinforcer; AS, Aversive stimulus; GI, Giving Information; WB, Well-Being; AI, Asking for Information; TB, Target Behavior; FI, Following Instructions; PB, Problem Behavior; T1, Treatment phase 1; T2, Treatment phase 2; *p* < 0.01.

**Table 3:** Three- and four-term pattern analysis using THEME.

Pattern type	Number of patterns		
*Three-term pattern*	Evaluation	T1	T2
CD-GI-R+	15	–	–
CD-TB-R+	–	-	95
ID-TB-CMO	9	15	-
CMO -TB-R+	31	35	66
D-CD-GI	9		–
WB-CD-GI	–	12	–
PB-AS-TB	–	–	10
*Four-term pattern*			
GI-DC-TB-R+	–	–	55
TB-CMO-TB R+	–	21	–
DC-TB-R + -PB	–	–	9

CD, Clinical Discriminative Stimulus; ID, Instructional Discriminative Stimulus; CMO, Conditioned Motivating Operation; R+, Positive Reinforcer; AS, Aversive stimulus; GI, Giving Information; TB, Target Behavior; PB, Problem Behavior; T1, Treatment phase 1; T2, Treatment phase 2; *p* < 0.01.

Table 3 shows the three- and four-term patterns in each phase. The most repeated three-term patterns involve *Target Behaviors* and *Positive Reinforcers* (e.g., DC TB R+; CMO TB R+). Our analysis reveals similar data with four-term patterns. The most repeated patterns involve *Target Behaviors* and *Positive Reinforcement* (e.g., GI DC TB R+; TB CMO TB R+).

### Comparison between both software

Table 4 shows a comparison between the patterns with two variables detected with GSEQ and THEME. Specifically, 59% (i.e., 17/29) of the identified patterns were detected by both methods of detection. While 31% (i.e., 9/29) were detected only by THEME, 10% (i.e., 3/29) were detected by GSEQ.

**Table 4:** Two-term patterns detected in each software.

Antecedent patterns	Evaluation	T1	T2
CD-GI	GSEQ and THEME	GSEQ and THEME	GSEQ and THEME
CD-WB	–	THEME	THEME
CD-TB	–	–	THEME
CD-AI	–	–	THEME
CD-PB	–	–	THEME
ID-FI	–	GSEQ	GSEQ
ID-TB	GSEQ and THEME	GSEQ and THEME	–
CMO-TB	GSEQ and THEME	GSEQ and THEME	GSEQ and THEME
CMO-AI	–	–	THEME
Consequent patterns			
TB-R+	GSEQ and THEME	GSEQ and THEME	GSEQ and THEME
TB-CMO	THEME	–	GSEQ and THEME
WB-R+	GSEQ and THEME	GSEQ and THEME	–
PB-AS	GSEQ	GSEQ and THEME	GSEQ and THEME
AI-CMO	–	–	GSEQ and THEME
GI-R+	–	–	THEME
FI-R+	–	–	THEME

Antedecent patterns are formed by clinician’s behaviors followed by the client’s behaviors; Consequent patterns are formed by clinician’s behaviors followed by the client’s behaviors; CD, Clinical Discriminative Stimulus; ID, Instructional Discriminative Stimulus; CMO, Conditioned Motivating Operation; R+, Positive Reinforcer; AS, Aversive stimulus; GI, Giving Information; WB, Well-Being; AI, Asking for Information; TB, Target Behavior; FI, Following Instructions; PB, Problem Behavior; T1, Treatment phase 1; T2, Treatment phase 2; *p* < 0.01.

### Visual distribution of the patterns

**Figure 3:**
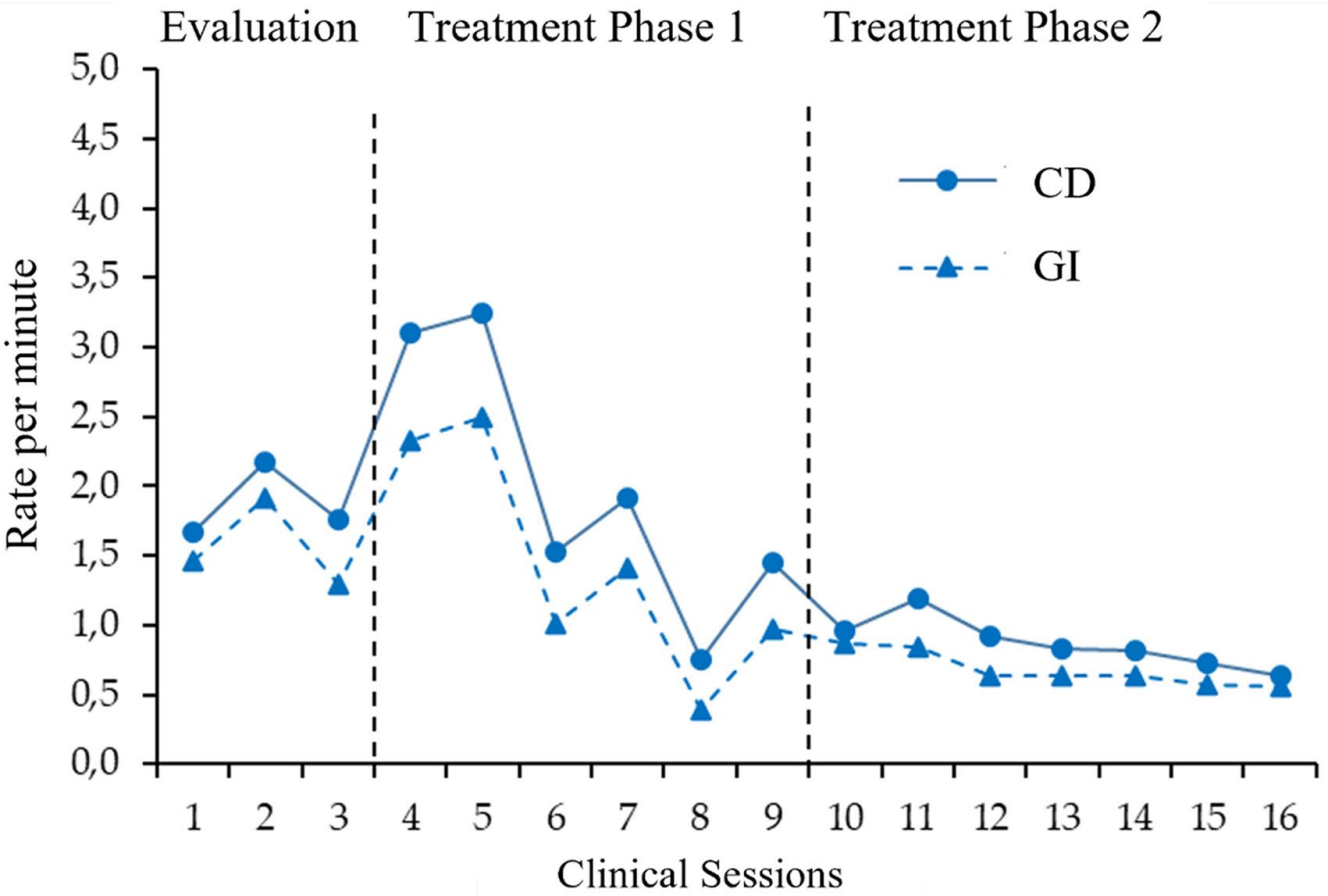
Clinical Discriminative and Giving Information distribution though sessions. This figure displays the rate per minute covariation between two sequentially correlated behaviors through different clinical sessions. Specifically, here we can see how the clinician’s behavior Clinical Discriminative Stimulus rate matches with the client’s behavior Giving Information during the treatment. CD, Clinical Discriminative Stimulus; GI, Giving Information; T1, Treatment phase 1; T2, Treatment phase 2.

**Figure 4:**
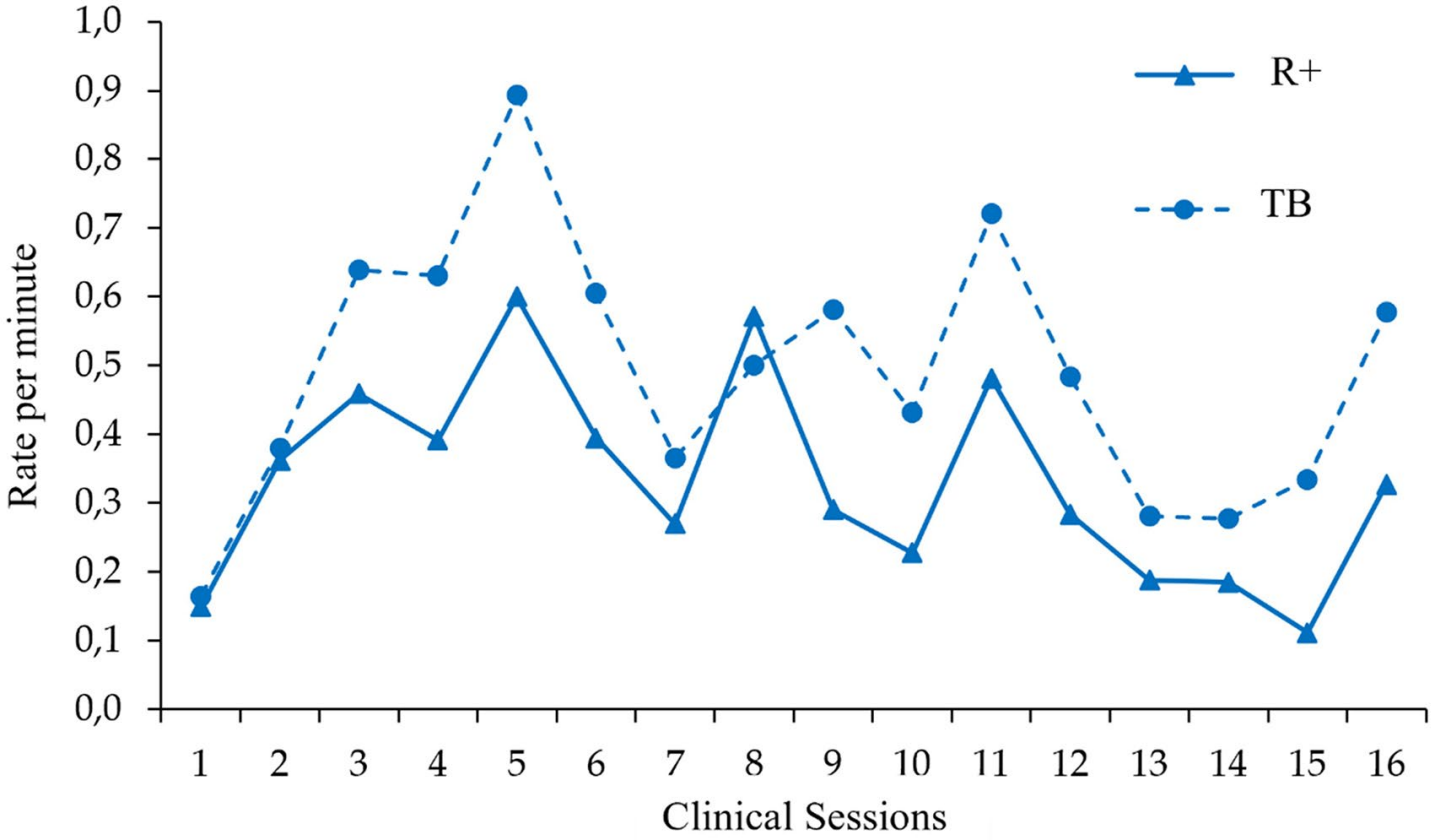
Target Behavior and Positive Reinforcer distribution though sessions. This figure displays the rate per minute covariation between two sequentially correlated behaviors through different clinical sessions. Specifically, here we can see how the clinician’s behavior Positive Reinforcer rate matches with the client’s behavior Target Behavior during the treatment. R+, Positive Reinforcer; TB, Target Behavior.

Pattern detection has indicated which variables are moment-to-moment correlated in verbal interaction. These results have allowed us to analyze how these variables change along with the psychological treatment. Figures 3, 4 show the distribution, through a clinical session, of the variables that are present in the most repeated patterns. These figures have been useful for visual analysis of the covariation of correlated variables.

For example, Figure 3 displays the evolution of verbalizations with *Clinical Discriminative* functions among different treatment phases and how are they followed by *Giving information* verbalizations of the client. As we will discuss these visual inspections could help practitioners to evaluate the clinical relationship or the efficacy of the clinical intervention.

### Effect sizes

Both sequential analysis and pattern analysis indicate positive correlations between the client’s *Target Behaviors* and the therapist’s *Positive Reinforcers*. To assess the effect of these *Positive Reinforcers* we have calculated the effect size in the client’s *Target Behaviors* (e.g., *Positive Reinforcers* should increase the likelihood of emission of the behaviors they follow). In this case, the effect size index indicates that the treatment had a moderate effect on increasing the *Target Behaviors* (NAP = 0.67; Standard Error: 0.21; 95% CI: [0.32–0.89]).

## Discussion

The aim of the current article was three-fold. First, we have highlighted the potential relevance of pattern analysis methods in the study of verbal interaction in psychological interventions and we have presented the reasons why these types of analysis could help in the development of the next generation of process analysis in clinical research. Second, we have conducted an observational proof-of-concept study to analyze the verbal interaction in a single-case design using two methods of pattern analysis (e.g., GSEQ and THEME). Using sequential analysis and more complex pattern detection algorithms we could identify more than 25 interaction patterns between a clinical psychologist and his client in different phases of the psychological treatment. These interaction patterns were visually displayed through different sessions, and the effect size of the treatment was measured for the *Target Behaviors* of the client. Third, we have compared the results yielded by these two methods of pattern analysis. In this section of the article, we discuss methodological implications in the search for hidden patterns of interaction in clinical settings, the clinical implications of our results, the limitations of our work, and the main conclusions.

### Methodological implications

We have compared the performance of GSEQ and THEME software in the detection of patterns in verbal interaction. They provided similar results in the detection of sequential patterns of two variables; approximately 60% of these patterns were detected by both (see Table 4). Although 60% could seem a low percentage of agreement, they detected in the most repeated patterns and there was not any difference in the main repeated patterns. THEME detected 9 patterns, in different moments of the treatment, that GSEQ did not. It seems that THEME could be more sensible to patterns with lower frequencies than the GSEQ. Thus, if the purpose is to explore hidden patterns of interaction, THEME is more useful in this regard. But if the aim is to detect the most significant patterns of interaction between two variables, both perform equally. At this point, we would like to bring attention to the negative correlations exposed by GSEQ. Both methods could be useful to assess treatment integrity, but the negative correlations give us extra information. It gives us not just about the apparition of psychologist behavior when a specific client’s behavior occurs, but also the absence of a certain psychologist behavior after the client’s behavior. For example, thanks to this data we could detect that his psychologist has not presented any *positive reinforcer* after a *problem behavior* of the client (see Figure 2).

One of the main differences between both software is the detection of patterns constituted by more than two variables. THEME showed to be powerful enough to automatically detect these patterns. While with GSEQ is possible to calculate different lag distances (e.g., Lag +2, Lag +3, etc.), the interpretation could lead to erroneous conclusions, because it did not include the variables that are inside of this correlation, and the software simply correlate two variables that are in a specific distance. That is not the case with THEME, it automatically has detected patterns formed by three and four correlated variables. This feature is essential to the study of verbal interaction because it has been useful to detect the repetition of structured patterns that imply a use by the psychologist of three- and four-term contingencies. In this study, we have detected 7 three-term patterns and 3 four-term patterns. This detection imply that we have increased the precision of this analysis, if we compare it with previous research on this topic (e.g., Calero-Elvira et al., 2013). Thus, the combination of both methods seems to be suitable to detect negative correlations and to detect complex patterns (i.e., three- and four-term contingencies).

### Clinical implications

As we have discussed, the use of sequential analyses and pattern recognition algorithms to analyze the verbal interaction between psychologist and client could help us to study how the learning processes are set in motion in psychological interventions. The observational data analysis of this paper has permitted us to have a closer look at this interaction and to describe how this interaction has occurred during the treatment. Among the results, we would like to discuss the clinical relevance of several patterns. Specifically, we were able to detect that *Clinical Discriminative Stimulus* correlates significantly with the client’s *Giving Information* behavior in all phases of the case, showing antecedent discriminative control by the psychologist of the client’s *Giving Information* behavior. Figure 3 shows how the rate of these behaviors changes similarly during treatment. These data could inform us that the psychologist has a good therapeutical relationship with the client, due to the positive correlation between these two variables. The sequential antecedent analysis indicates that *Following Instructions* correlates positively with the occurrence of *Instructional Discriminative Stimulus*, but this only occurs in the treatment phases and it is not found in the assessment phase. These differences between the evaluation phases could be explained by the positive correlation found in the evaluation phase between *Target Behaviors* and *Instructional Discriminative Stimulus*. This contingency also seems to occur to a lesser extent in the first treatment phase and coincides with the lower correlation between *Following Instructions* and *Instructional Discriminative Stimulus* in the treatment phases. *Instructional Discriminative Stimulus* may have correlated in the assessment with topographies of the *Target Behaviors*, but this study is not sensitive to such topographies. Also, we have detected a correlation between *Target Behaviors* and the *Conditioned Motivating Operations.* This correlation could be clinically explained by the conditioning function of the *conditioned motivation operations*. These verbalizations have the purpose of changing the client’s motivational value of some stimulus or activities. If the clinician states a verbalization with this function and the client agrees, it is probable that this agreement could be coded as a *target behavior*. Also, if the clients state a *Target Behavior*, the clinician could explain more details about why the client is right or relate it to their therapeutic goals, so these verbalizations could have a motivating function. Moreover, this relationship between *Target Behaviors* and *Conditioned Motivating Operations* also appears to have a key role in patterns with more complex structures (i.e., three- and four-term patterns; see Table 3). Due to this correlation having the potential impact of changing the client’s value of events, it could have a significant role in the clients’ behavioral change outside the clinical context and it should be studied in detail in future studies.

Also, P*ositive Reinforcers* correlate positively with *Target Behaviors*, in the three phases of the case; with verbalizations of *Well-Being*, in the assessment and the first part of the treatment; and with *Following Instructions*, in the first part of the treatment. This could mean that these behaviors are under a schedule of positive reinforcement applied by the psychologist. At the same time, positive reinforcers do not correlate with problem behaviors in the assessment and correlate negatively in the treatment statements. This could indicate that the therapist identifies problem behaviors once treatment has already begun and he does not apply positive reinforcement schedules. In contrast, *Aversive stimuli* correlate positively with *Problem Behaviors*. Thus, that could mean that these behaviors are under a reduction procedure applied by the psychologist.

As with *Conditioned Motivating Operations*, *Positive Reinforcer* correlates with *Target Behaviors* even in three- and four-term patterns. CMO-TB-R+; CD-TB-R+; GI-DC-TB-R+; and TB-CMO-TB-R+. Figure 4 shows how *Positive Reinforcers* and *Target Behaviors* covary through the treatment. It seems that the psychologist was using reinforcing contingencies that include *Target Behaviors*. The theoretical effects of these contingencies should imply an increment of *Target Behavior* in session. Calculating the effect size in the increment of the *Target Behaviors*, we tried to indirectly assess whether this procedure affects these behaviors. Results showed us that the effect size was moderate. Other potential variables could influence this class of behaviors, further experimental analysis should confirm the relationship that we have detected in this study.

### Limitations

We have found problems in the use of the GSEQ and THEME software with raw data obtained using *The Observer XT 12* to the GSEQ and THEME software. Both software could develop techniques to import the results from observational software with ease. Moreover, as we discussed, we have found that the THEME’s complex pattern detection performance is superior to the GSEQ performance in the same task. Although THEME seems to be sensible to patterns with low frequency, this is more a challenge in the interpretation of the results to the researcher than a limitation of the software. Also, we have not analyzed all the patterns that THEME has detected, we impose some pre-analysis requirements. Without these requirements, patterns reported by THEME could increase.

Also, the observational coding of events was not automated. The functional definitions of the variables have increased the complexity of the observational coding. In this sense, the reproducibility of the results is compromised, because this methodology of research is time-consuming, and it requires observers with high standards of training. Although the complexity is an issue, this analysis could be benefited from the inclusion of qualitative data on the verbalizations.

Finally, the results of this study are merely tentative and further experimental analyses need to be conducted to fully study the patterns of interaction that are occurring in the psychological interventions. Also, this experimental control could help to better analyze the effect size of the treatment. The influence of external variables could have affected the behavior of the client. Moreover, we recognize that this study could have all the potential limitations of the single-case research. For example, the results derived and analyzed in this study are not representative of the clinical interaction in all clinical cases, and results could be biased by several factors (e.g., culture, psychologist’s training, client’s psychological problems, etc.). But, despite all these limitations, we believe in the exploratory value of this paper, it could be useful for the development of new perspectives and methodologies for the study of processes in clinical psychology.

## Conclusion

The study of the processes underlying therapeutic change is essential to optimize psychological treatments. The identification of patterns of verbal interaction during therapy is a valuable step in understanding how the processes that make psychological treatments work are set in motion. GSEQ and THEME software have proven to be able to detect those patterns in verbal interaction. THEME has proven to be more powerful in detecting complex interaction patterns and more sensitive in detecting low-frequency patterns, yet both have detected predominant patterns in verbal interaction that may underlie clinical change. This implies that pattern recognition methods could be seen as a promising alternative to studying behavioral change processes in psychological treatments. These methods combined with single-case designs and the development of new recently developed effect sizes for this type of studies (e.g., Pustejovsky, 2018), could have a unique impact on the development of clinical research in the forthcoming years.

## Data availability statement

The datasets presented in this study can be found in online repositories. The names of the repository/repositories and accession number(s) can be found at: https://osf.io/ezhmd/?view_only=dbe50c17e90a419e93c87116703ceb27.

## Ethics statement

The studies involving human participants were reviewed and approved by Autonomous University of Madrid. The patients/participants provided their written informed consent to participate in this study.

## Author contributions

All authors listed have made a substantial, direct, and intellectual contribution to the work and approved it for publication.

## Funding

This research was supported by grant PSI2016-76551-R from the Ministerio de Economía, Industria y Competitividad of the Spanish Government.

## Conflict of interest

The authors declare that the research was conducted in the absence of any commercial or financial relationships that could be construed as a potential conflict of interest.

## Publisher’s note

All claims expressed in this article are solely those of the authors and do not necessarily represent those of their affiliated organizations, or those of the publisher, the editors and the reviewers. Any product that may be evaluated in this article, or claim that may be made by its manufacturer, is not guaranteed or endorsed by the publisher.

## Footnotes

^1^To analyze the relationship between the early functional conceptualizations of the verbal behavior to the development of empirically supported psychological treatments is beyond the scope of this paper, but it can be found elsewhere (see Hayes, 2004; Froxán-Parga et al., 2018; Hayes and Hofmann, 2018; Barnes-Holmes et al., 2020).
